# A case of modular phenotypic plasticity in the depth gradient for the gorgonian coral *Antillogorgia bipinnata* (Cnidaria: Octocorallia)

**DOI:** 10.1186/s12862-017-0900-8

**Published:** 2017-02-17

**Authors:** Iván Calixto-Botía, Juan A. Sánchez

**Affiliations:** 1 Department of Animal Ecology and Systematics, Justus Liebig Universität, Heinrich-Buff-Ring 26-32 IFZ D-35392, Giessen, Germany; 2 Laboratory of Biología Molecular Marina-Biommar, Department of Biological Sciences-Faculty of Sciences, Universidad de los Andes, Carrera 1E No 18A – 10, P.O. Box 4976, Bogotá, Colombia; 3 Marine Sciences, International Giessen Graduate Centre for the Life Sciences (GGL), Justus Liebig Universität, Giessen, Germany

**Keywords:** Phenotypic plasticity, *Antillogorgia bipinnata*, Reaction norm, Octocoral, Depth cline

## Abstract

**Background:**

Phenotypic plasticity, as a phenotypic response induced by the environment, has been proposed as a key factor in the evolutionary history of corals. A significant number of octocoral species show high phenotypic variation, exhibiting a strong overlap in intra- and inter-specific morphologic variation. This is the case of the gorgonian octocoral *Antillogorgia bipinnata* (Verrill 1864), which shows three polyphyletic morphotypes along a bathymetric gradient. This research tested the phenotypic plasticity of modular traits in *A. bipinnata* with a reciprocal transplant experiment involving 256 explants from two morphotypes in two locations and at two depths. Vertical and horizontal length and number of new branches were compared 13 weeks following transplant. The data were analysed with a linear mixed-effects model and a graphic approach by reaction norms.

**Results:**

At the end of the experiment, 91.8% of explants survived. Lower vertical and horizontal growth rates and lower branch promotion were found for deep environments compared to shallow environments. The overall variation behaved similarly to the performance of native transplants. In particular, promotion of new branches showed variance mainly due to a phenotypic plastic effect.

**Conclusions:**

Globally, environmental and genotypic effects explain the variation of the assessed traits. Survival rates besides plastic responses suggest an intermediate scenario between adaptive plasticity and local adaptation that may drive a potential process of adaptive divergence along depth cline in *A. bipinnata*.

**Electronic supplementary material:**

The online version of this article (doi:10.1186/s12862-017-0900-8) contains supplementary material, which is available to authorized users.

## Background

Phenotypic plasticity has been defined as the natural capacity of an organism to react to a phenotypic change in form, state, movement or activity rate in response to environmental variation [1]. In the past century, phenotypic plasticity was largely considered a barrier to speciation: if there is no need for genetic change to adapt to the environment (masking the genotype for negative selection), then the process of adaptive genetic divergence will be hindered [2, 3]. However, the potential role of adaptive plasticity in promoting speciation has been suggested in some cases where it can contribute to niche diversification and further evolutionary change [1, 4–6]. Phenotypic and genetic accommodation, the Baldwin effect, and the Waddington’s genetic assimilation have been proposed to explain environmental-induced changes fixed in the genome and susceptible to promote a speciation process [7, 8].

Reciprocal transplant experiments consist of transferring phenotypic variants to the opposite environments and are a practical and cost-effective approach to testing phenotypic plasticity. In marine systems, variation related to environmental heterogeneity has been specially studied along depth gradients, which can vary in light intensity, wave exposure and nutrient concentration. In corals, most studies assessing plasticity and genetic adaptation have found that adaptive divergence corresponds to depth gradients. One of the first experiments using a common garden approach on corals was done with *Orbicella annularis* and *Siderastrea siderea*, in four reef habitats and detected high phenotypic plasticity in response to the transplanted habitat [9]. In another study, shallow and deep morphotypes of *Eunicea flexuosa*, a Caribbean gorgonian, exhibited low phenotypic plasticity of sclerites and a strong genetic divergence signal [10]. Finally, using reciprocal transplants of *Seriatopora hystrix*, a case of adaptive plasticity was detected in response to light conditions and adaptive divergence along the depth gradient [11, 12].

Some Caribbean corals, including the feather-like gorgonian coral *Antillogorgia bipinnata* (Verrill 1864), possess broad environmental preferences, sympatric distributions, and contain highly plastic species complexes that are likely undergoing incipient ecological speciation processes [13–15]. *A. bipinnata* is distributed along coral reefs from 1 to 45 m deep in Panama, Belize, Bahamas, Florida, Colombia, but is absent on the Eastern side of the Western Atlantic. Along with this bathymetric gradient, the species varies phenotypically, including variation in size, coloration, sclerite form and branching pattern. Three basic morphotypes can be recognised over a depth cline, the ‘*deep morphotype*’, ‘*typical morphotype*’ and ‘*bushy morphotype*’ or ‘*kallos*’ [15], the latter (*A. kallos* Bayer) described as a distinct species [16, 17]. In some coral reefs (such as Panama) including at depths as shallow as 7 m, all three morphotypes can be present where there is an abrupt change in depth and reef slope, suggesting that this physiological challenge promotes an adaptive morphological response.

To identify the contribution of plasticity to trait variation in corals emerging from the colonial structure, a reciprocal transplant experiment was carried out between the deep and bushy morphotypes of *A. bipinnata* from two locations in Bocas del Toro, Panama. With this research, we measured modular traits related to bathymetric adaptation to detect the genotypic and environmental components involved in colonial structure variation between morphotypes of *A. bipinnata*. These data deepen our understanding of the evolutionary mechanisms and patterns of diversification for a remarkable number of species from this subclass that show marked phenotypic variation related to environmental gradients [17, 18].

## Methods

### *Antillogorgia bipinnata*

Based on the molecular and morphological evidence, *Antillogorgia bipinnata* (Verrill) has been recently reassigned from the genus *Pseudopterogorgia* [19]. Populations of the complex *A. bipinnata-kallos* are clustered in reefs in the Southern Caribbean, the Mesoamerican Reef, with Panama and Belize populations closely related [20], and the Florida-Bahamas region. Colonies of the ‘deep’ morphotype are larger, with longer principal axes, secondary branches, and internodal lengths, but fewer secondary branches in contrast to the ‘bushy’ morphotype. These traits emerge from their modular organisation, with the polyp as an iterative unit (sensu stricto) and branch as derived modular units [21, 22]. Thus, differences in the architectural pattern between morphotypes shape the distribution and polyp density across the colony. Colony architecture has a feasible role in nutrient capture, overall photosynthetic rate, physical stress, and can be directly related to adaptive responses to environmental variables in the depth cline.

### Study Area

**Fig. 1:**
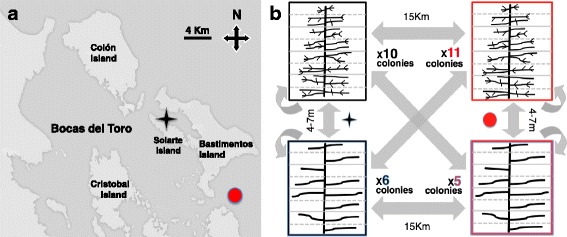
Locations and design of the reciprocal transplant experiment. **a** Map of Bocas del Toro, Panama, signalling the localities of Hospital point (*black star*) and Crawl key (*red circle*) (basemap from https://google.com/maps/). **b** Experiment design with arrows indicating the direction of transplants between habitats and localities with *curved arrow* for native controls. Eight segments per colony were used to get a fully crossed and replicated design

The experiment was conducted at two localities in the northern Panamanian Caribbean, Hospital Point and Crawl Key, Bocas del Toro (Fig. 1a). These locations exhibit some environmental differences; Hospital Point is a protected reef with abundant suspended particles and low water motion compared to Crawl Key. 15 km away, Crawl Key is an exposed reef flat, characterised by eroded coral skeletons and greater light penetration. Shallow habitats are characterised by coarser sand with corals typically established on hard surfaces, such as rocks. In contrast, in deep habitats, the slope is lower with greater sediment perturbation. Data for temperature and illuminance (total luminous flux per unit area) were collected using HOBO temperature and light data loggers (Hobo Water Temp Pro, Onset Computer Corp., Bourne, Mass). In the shallow habitat at both localities, the temperature ranged between 26–31 °C in comparison to 26–30 °C for deep habitats. Illuminance strongly varied between habitats with the greatest variation in shallow habitats of Crawl Key (0–35800 lux), and Hospital Point (0–30300 lux). In deep habitats, illuminance varied from 0–14400 lux for Crawl Key and 0–2900 lux at Hospital Point.

### Establishing reciprocal transplants and data collection

To assess the response of modular traits after transplantation, we took advantage of the overcompensation phenomenon in *A. bipinnata*, a fast apical branching response following injury [23]. Fragments approximately 30 cm long were cut from 32 healthy colonies of the bushy and deep morphotypes at Crawl Key and Hospital Point. The 16 colonies collected from each locality were at least 12 m apart from each other, a total of 21 bushy and 11 deep morphotypes. In a fully factorial design, we transplanted 256 coral segments across shallow (2–3 m) and deep (7–8.5 m) habitats in both locations, one control kept in the native habitat and three experimental units in two replicate groups (Fig. 1b). Segments from the colony source were randomly assigned to destinations. To firmly attach the explants, each was fixed to a piece of PVC-pipe with PVC-clamps [23]. To collect data on focal colonial traits, we took pictures (PowerShot G12, Canon®) following initial transplantation, on July 13–14, 2011, and at the end of the experiment, on October 17, 2011, 13 weeks later. Segments were considered dead if high tissue loss was detected (>50%). A background grid (white acrylic board) with the scale was used for image correction and later measuring in the digital processing.

### Digital processing and statistical analysis

We used Adobe Photoshop® software (Adobe Systems, San Jose, CA) to set pictures to a single optical plane for perspective correction. ImageJ® software [24] was used for measuring the traits, by transforming the scale from pixels to metric units [21]. We measured modular traits: (1) the *vertical length variation* by measuring the variation in length of the main axis, (2) *horizontal length variation* by haphazardly selecting secondary branches (1688 branches, $\overline{x}=9.4$ per segment) and measuring the variation in length at both time points, and (3) *new branches promotion* generated after injury, in the growing apical segment as well as branches on old secondary branches.

Linear mixed-effects models (LMMs) were constructed to explain the variance in the traits measured between morphotypes and habitats. With this model, we assessed the effect of the response variables both separately and jointly over trait performances. Response variables (vertical length, horizontal length, and new branch promotion) were regressed on the predictors morphotype (two levels, bushy and deep) and target habitat (two levels, shallow and deep) as well as on their interactions. Location (Hospital Point and Crawl Key) was included as a random effect to account for environmental differences, as well as the internal variance of morphotypes (genotypes nested into morphotypes). The significance of the model terms was assessed using Akaike information criterion (AIC) and calculated with the “dredge” function [25]. AIC value below two suggests substantial evidence for the model, values between 3 and 7 means that the model has considerably less support, and values over ten indicates that the model is very unlikely [26]. Fixed variables from all models with AIC values below two, were examined for Beta, t-student and *p*-values to test for significant relationships. The significance values of Beta, t-student and *p*-values supported the analysis of AIC test for a better interpretation across each predictor assessed. In addition, the normality of data was examined with exploratory graphics of quantile-quantile plots.

A statistical significant morphotype effect indicates that genotype differences may explain the response. A significant target habitat effect on trait variation implies plasticity, indicating consistent variation in the trait with the environment. A significant morphotype by target habitat interaction indicates a genotype by environment effect on the response (G X E), where variation in the degree of plasticity between morphs is detected. A graphical approach to joint statistical analysis was performed using reaction norms of the three assessed traits using the median values (M) and Median Absolute Deviation (MAD) keeping in mind the asymmetric distributions of the resulting variance. Reaction norms for new branches promotions were constructed using a density of branches (number of branches over a length of the main axis) to get appropriate slopes in cases of null promotion. All statistical analyses were performed in R version 3.2.3 [27] using the *lme4* package [28].

## Results

Sixty one of the initial 256 segments were lost during photo recovery or analysis (e.g. it was not possible to identify the code of the colony, the metric references or to recognise some of the traits). From the remaining 195 explants, 16 died (*see* Additional file 1), giving a 91.8% of survival and providing a large enough sample size to assess fixed factors.

**Fig. 2:**
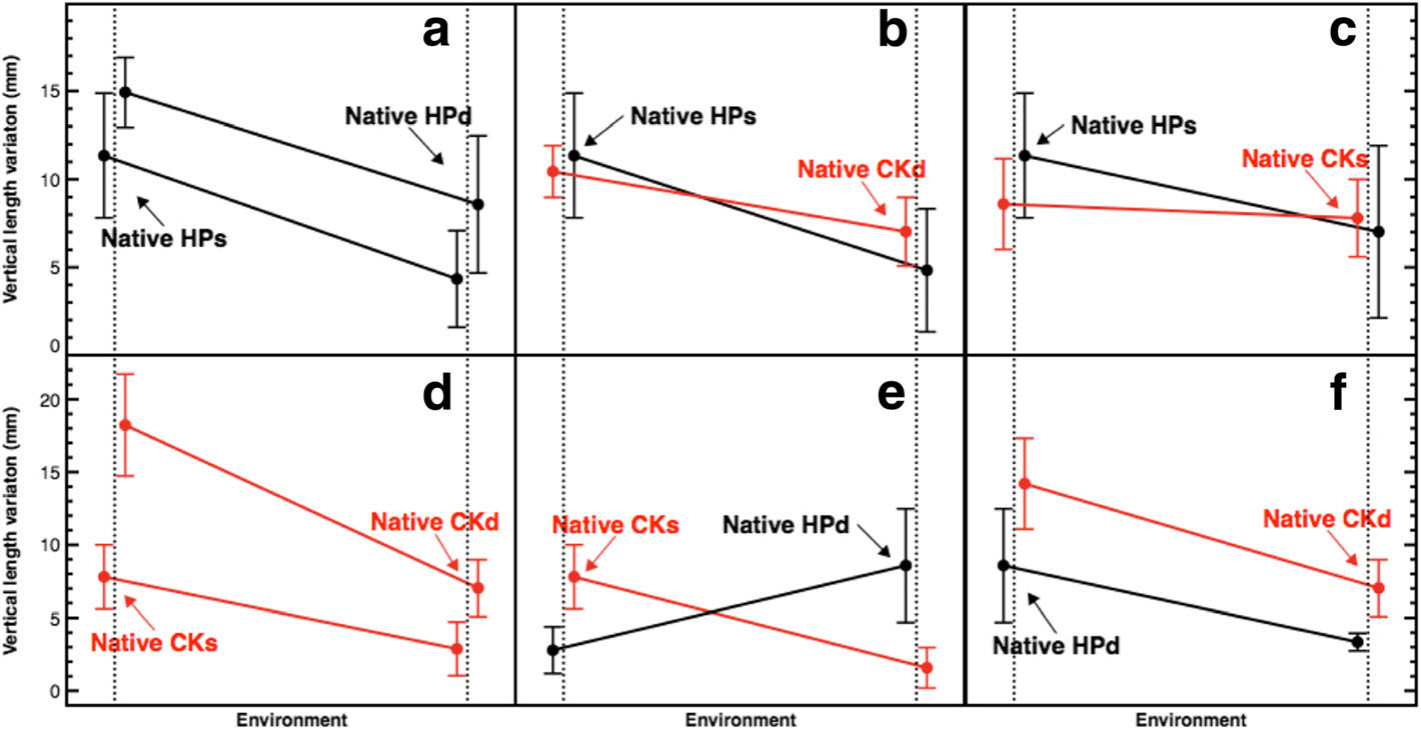
Reaction norms for vertical length variation in A*. bipinnata*. **a**-**f**, on the Y-axis is the vertical length variation in mm and on the X-axis the environment. Colours encode source locality of colonies: black for Hospital Point and red for Crawl Key. Data are laterally offset for improved visualisation. Dots represent the median magnitudes and bars the + − MAD (Median Absolute Deviation). HPs = Hospital Point shallow, HPd = Hospital Point deep, CKs = Crawl Key shallow, CKd = Crawl Key deep

LMM, significance test and reaction norms assessed signs to target habitat together with morphotype as the main explanatory variables of the variance for the three modular traits of *A. bipinnata*, i.e. plasticity pattern, as well as a genetic component, explains the variance 13 weeks after transplantation. Q-Q plots did not show overdispersion of data for any of the variables. Ranking of LMM highligted in each of the three variables target habitat and morphotype as general explanators of variance for AIC values below 2 (Table 1). Therefore, for the response variable vertical length variation, predictable terms based on AIC contained all terms except morphotype alone. Nevertheless, the significance test only indicated that length differed between deep and shallow habitats in the destination variable (β = −2.52 [SE = 1.09], t (d.f. = 71.9) = −2.30, *p*-value = 0.023). Reaction norms for vertical length variation (Fig. 2) show that higher values were common at shallow habitats, for example, the deep morphotype in shallow habitat at Crawl Key (M = 18.23, MAD = 3.51). Except for segments from deep habitat at Hospital Point that were transplanted to Crawl Key shallow habitat (Fig. 2e), the slopes between habitats were in the same direction and in some cases, close to parallel (Fig. 2a, d and f).

**Table 1:** Ranking of Linear Mixed-effects Model for three modular traits

Trait	Model	AIC	Δi	d.f.	Weight
Vertical length variation	TgHt, Mrph	673.3	0.00*	6	0.343
	TgHt	673.5	0.19*	5	0.311
	TgHt, Mrph, TgHt:Mrph	674.3	1.02*	7	0.205
	Mrph	676.4	3.07	5	0.074
		676.5	3.25	4	0.067
Horizontal length variation	TgHt, Mrph	580.1	0.00*	6	0.742
	TgHt, Mrph, TgHt:Mrph	582.4	2.25	7	0.240
	Mrph	587.8	7.66	5	0.016
	TgHt	592.3	12.15	5	0.002
		598.6	18.50	4	0.000
New branches promotion	TgHt	855.6	0.00*	5	0.427
	TgHt, Mrph	856.2	0.52*	6	0.329
	TgHt, Mrph, TgHt:Mrph	856.9	1.22*	7	0.232
		863.9	8.30	4	0.007
	Mrph	864.5	8.84	5	0.005

Each model incorporates both fixed- and random—effects terms in the linear predictor expression, from which the conditional mean of the response can be evaluated. *AIC* Akaike information criterion, *Δi* delta in AIC score with respect to the best model, *d.f.* degrees of freedom *TgHt* Target Habitat; Mrph, Morphotype. AIC values below two are marked with asterisks

**Fig. 3:**
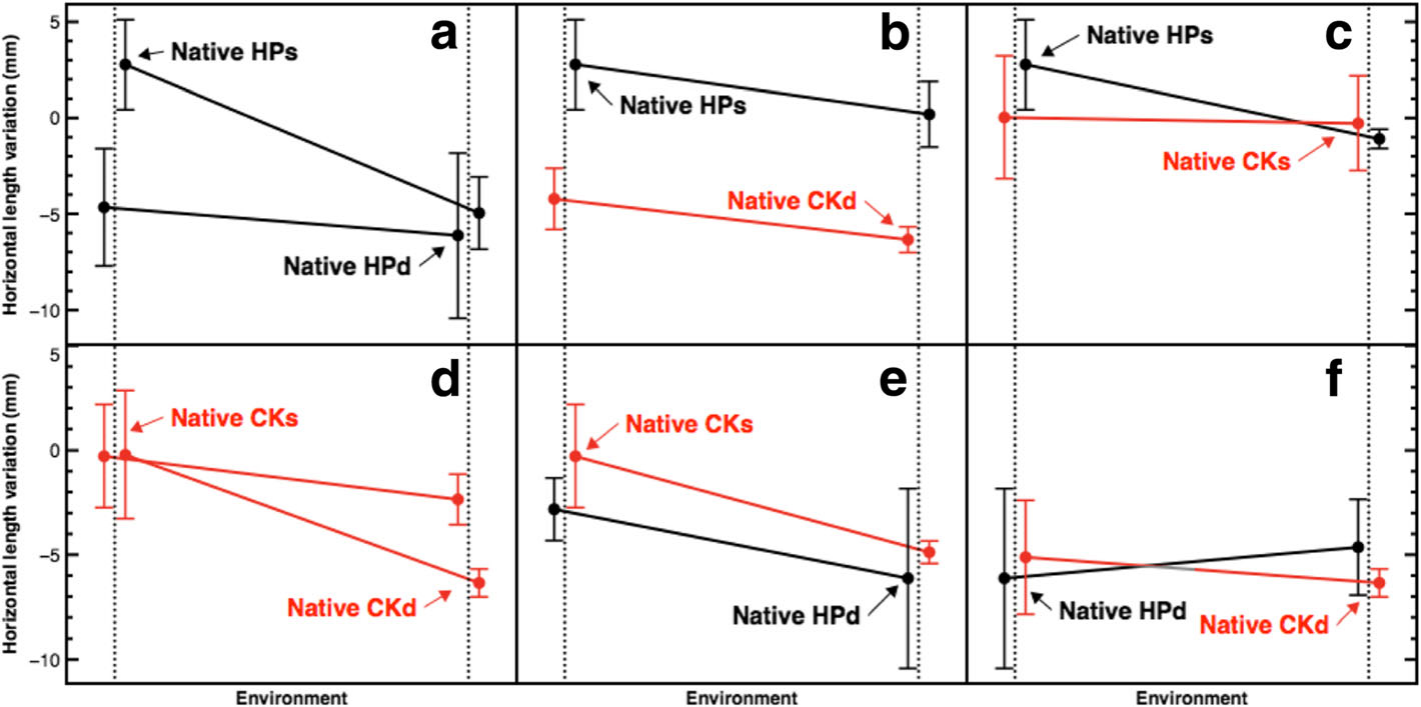
Reaction norms for horizontal length variation in *A. bipinnata*. **a**-**f**, on the Y-axis is the horizontal length in mm and on the X-axis the environment. Colours encode source locality of colonies: black for Hospital Point and red for Crawl Key. Data are laterally offset for improved visualisation. Dots represent the median magnitudes and bars the + − MAD. HPs = Hospital Point shallow, HPd = Hospital Point deep, CKs = Crawl Key shallow, CKd = Crawl Key deep

The highest ranked model for horizontal length variation based on AIC only contained the terms target habitat and morphotype. Horizontal length differed between deep and shallow destination (β = −2.54 [0.79], *p* = 0.001) along with to the type of morphotype (β = 3.24 [0.83], *p* = 0.0006). Compared to the positive values for vertical lengths, in most cases, horizontal lengths were more variable and reached negative values even for native explants, indicating loss of secondary branch tissue (Fig. 3). Higher horizontal growth values were evident in shallow habitats, with peaks in shallow natives from Hospital Point (M = 2.77; MAD = 2.34) compared to deep natives from the Crawl Key locality (M = −6.35, MAD = 0.67). Most slopes were in the same direction with the exception of Fig. 3f and nearly parallel as in Fig. 3b and e (involving transplants between habitats and locations).

**Fig. 4:**
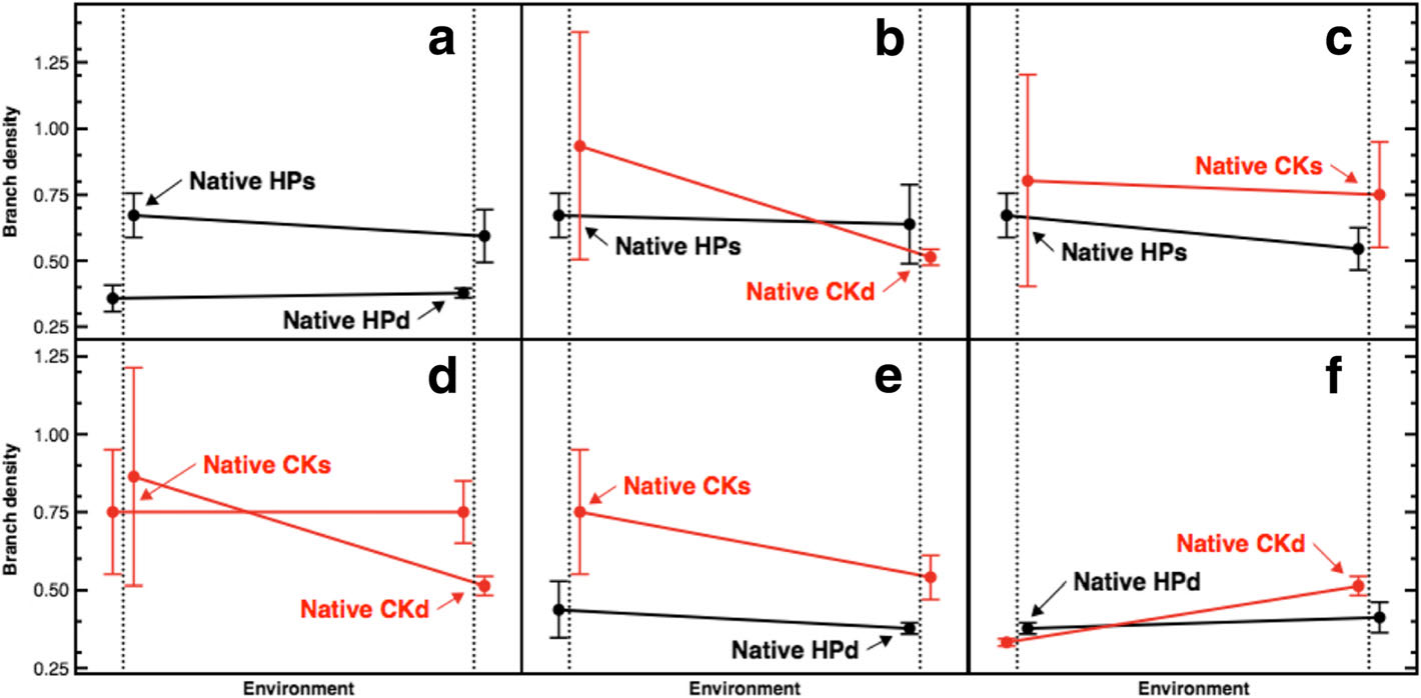
Reaction norms for branch density in *A. bipinnata*. **a**-**f**, on the Y-axis the branch density calculated over the length of the main axis and on the X-axis the environment. Colours represent source locality of colonies: black for Hospital Point and red for Crawl Key. Data are laterally offset for improved visualisation. Black and red dots represent the median magnitudes and bars the + − MAD. HPs = Hospital Point shallow, CKs = Crawl Key shallow, CKd = Crawl Key deep

Promotion of new branches was higher in shallow habitats compared to deep ones, as such the highest ranked model based on AIC contained the term target habitat (β = −2.52 [1.09], *p* = 0.023). Reaction norms using branch density (Fig. 4) show that a large number of branches were generated in the explants from Crawl Key deep to Crawl Key shallow (Fig. 4d; M = 0.86; MAD = 0.35) and to Hospital Point shallow (Fig. 4b; M = 0.93; MAD = 0.43) in comparison to deep controls of Crawl Key (M = 0.51; MAD = 0.03) and Hospital point (M = 0.37; MAD = 0.01) and even the shallow natives.

## Discussion

The LMM tests and reaction norms graphs for each of the traits indicate adaptive phenotypic plasticity and genetic variance in a classical genotype and environment model of phenotypic response [1, 29]. This is congruent with a recent evaluation of a reciprocal transplant assessing the sclerite trait on the same species [30]. The general trend of variation in the three traits tested resembled the native transplants performance. The survival rate of foreign transplants and the trend towards native values in the reaction norms indicated some grade of adaptive response [29, 31]. For bushy segments transplanted into deep habitats, a lower vertical and horizontal growth and fewer new branches were found, compared to the general response of foreign deep segments in shallow habitats at both localities. Even when the standard errors were high, which could be typical for this kind of architectural organisation [21] the trait data is suggestive of adaptive phenotypic plasticity, i.e., slopes trend similarity [32, 33]. Non-parallel reaction norms within localities indicate even more strongly that plastic responses were similar but not identical, suggesting that ecological and genetic divergence involved in the different responses, where there is a large habitat influence but the phenotypic responses occur in different ways over the bushy and deep morphotypes [1, 34].

The vertical and horizontal length variation for most transplants was positive in the shallow habitats, indicating greater tissue growth, and negative in deep habitats, indicating lower tissue growth. In deep habitats, this was particularly true for horizontal branches, which experienced null growth and even tissue loss. Greater growth in native controls in shallow habitats compared to natives from deep habitats could be counterintuitive since longer main and secondary branches characterise deep morphotypes. However, colony growth analysis in *A. elisabethae*, a closely related species with the same colonial architecture, have shown that initial branching growth is greater in small colonies than in larger ones with a drastic reversion in time [21]. Thus, it is possible that growth in the *A. bipinnata* transplants could be explained by an initial difference in response to habitat after injury with an insufficient time to display such a reversion in branch growth behaviours.

At the same time, lost tissue (i.e. dead polyps) was most common on horizontal branches of segments situated in deep habitats, which could indicate that environmental conditions in these locations are stronger drivers for adaptation than shallow environmental conditions, as supported by the positive controls. One of these conditions is the higher level of sedimentation in deep habitats, which decrease the light intensity and may compromise the zooxanthellae photosynthetic process. This may be especially true at Hospital Point, which differed from Crawl Key by 11500 lux. Consequently, the lower abundance of colonies in deep environments support a range edge of morphotype performance [35], where it is possible that a controlled variable such as injury could act as a stressor in differential grades.

Different magnitudes of genotypic and environmental effects appear to occur across the three variables, which could be explained by different environmental drivers affecting each modular trait. The most conspicuous plastic response was new branch promotion, as statistics and reaction norms indicate. Reaction norms resembled the native ones from shallow habitats, where bushy morphotypes have the higher density of secondary branches on shorter main axes. In particular, the shallow habitat at Crawl Key had the most positive values for branch promotion, reaching as high as 127 new branches in only one deep morphotype segment (Fig. 4b, d). By contrast, there was almost no branch promotion from the two localities in the Crawl Key deep habitat. Since number of branches is a trait positively correlated to wave exposure and currents in gorgonians [21], high physical disturbance in shallow versus deep habitats at Crawl Key compared to low water motion at both depths at Hospital Point may explain this result. Similarly, in this group of corals, the light intensity is positively correlated with investment in branching. Therefore, in shallow habitats branching focused on secondary branches while in deep habitats the growth is focused on height, in order to increase light exposure [36].

These types of responses point to plastic properties in colonial organisms such as gorgonians, which could be expected to be more flexible in traits at a modular scale level [22, 37]. To understand the impact of modular properties on species evolability requires in addition to assessing canalization capacity, identifying other components, such as the genetic fixation mechanisms and cost-benefit trade-offs. At the same time, it could be advisable to assess life history attributes, such as game to genesis, number of reproductive cycles/years or larval production, which can serve as better proxies for fitness, even in challenging systems, such as corals [38].

## Conclusions

Immigrant inviability, proposed as a key element in reproductive isolation between morphotypes in the depth cline [39, 40], does not appear to be the main force in the evolution of phenotypic divergence in *A. bipinnata*. Instead, due to 83.3% of survival of transplanted segments in a previous transplant experiment [30] and 91.8% survival in addition to environmental and genotype effects found in this study, an intermediate scenario between adaptive plasticity and local adaptation seems to be being carried out in this species*.* Additional molecular evaluation of the genetic basis of modular traits divergence could be enlightening, keeping in mind that preliminary neutral genetic variation among *A. bipinnata* morphotypes in Panama seems to be negligible [20]. Assessing adaptive genetic divergence coupled with a functional background of positive selection signals could provide an integral perspective to test the current hypothesis of an incipient ecological speciation mediated by modular plasticity mechanisms in *A. bipinnata*.

## Additional file

Additional file 1: Raw data of the reciprocal transplant for two locations and two depths in *Antillogorgia bipinnata* (Cnidaria: Octocorallia). Growth data for each branch in the two times are in the same row. Photo_Code description follows the next example for 3459_AJ: 3459 unique number; A is the replicate (A or B); J (June) or O (October). (XLS 223 kb)
